# Aplicaciones y retos de ChatGPT en la medicina de laboratorio

**DOI:** 10.1515/almed-2025-0158

**Published:** 2025-12-08

**Authors:** Zhili Niu, Xiandong Kuang, Juanjuan Chen, Xin Cai, Pingan Zhang

**Affiliations:** Servicio de Análisis Clinicos, Institute of Translational Medicine, Renmin Hospital of Wuhan University, Wuhan, Hu Bei Province, Hubei, China

**Keywords:** ChatGPT, *chatbots*, laboratorio clínico, ventajas, desventajas

## Abstract

La rápida evolución de la inteligencia artificial ha permitido desarrollar *chatbots* con un enorme potencial en campos como el de la medicina, especialmente en el laboratorio clínico. Realizamos un análisis sistemático de las ventajas e inconvenientes que supone la utilización de *chatbots* en este campo, profundizando en sus posibles aplicaciones para el diagnóstico de enfermedades. La fiabilidad y veracidad científica de los *chatbots* se ven determinadas por diversos factores, entre los que se encuentran la calidad de los datos, los sesgos de los modelos, la protección de la privacidad, y los requisitos de retroalimentación del usuario. Sin embargo, el marco jurídico existente, como la Ley sobre inteligencia artificial (IA) de la UE, no garantiza por sí solo la veracidad y fiabilidad de los contenidos, por lo que no podemos depender únicamente del mismo, haciendo necesario el empleo de dos herramientas de evaluación, METRICS y CLEAR, herramientas diseñadas para evaluar de manera integral la calidad de la información relacionada con la salud generada por IA.

## Introducción

La inteligencia artificial (IA) fue conceptualizada por primera vez en 1950 por la pregunta fundamental planteada por Alan Turing “¿Pueden pensar las máquinas?”, que sentó los fundamentos teóricos de este campo de conocimiento [1]. El término “inteligencia artificial” fue oficialmente acuñado en 1956 por John McCarthy et al. en la Conferencia de Dartmouth, dando así lugar al nacimiento de la IA como una disciplina científica propia [1].

La IA abarca un amplio espectro, desde la inteligencia artificial general (IAG), que trata de replicar la inteligencia humana, hasta la inteligencia artificial estrecha (IAE) o débil, centrada en tareas específicas. En un principio, la IA empleaba algoritmos basados en reglas, como las reglas “si … entonces”, siendo estos sistemas no adaptativos aún empleados actualmente en muchos entornos clínicos. Sin embargo, los avances tecnológicos han permitido desarrollar métodos más sofisticados de IA, entre los que se encuentran los algoritmos de aprendizaje automático (ML, del inglés, *machine learning)* y de aprendizaje profundo (DL, del inglés*, deep learning)*, mejorando así el análisis de datos, el reconocimiento de imágenes y la predicción de enfermedades en el diagnóstico clínico.

Como resultado de la automatización gradual del laboratorio clínico, se generan a diario enormes volúmenes de datos de extrema complejidad, sobrepasando la capacidad de procesamiento del cerebro humano, y haciendo cada vez más necesaria la utilización de la IA. En el laboratorio clínico, la IA es capaz de analizar datos estructurados de gran calidad, lo que ha revolucionado los métodos diagnósticos [2]. Los modelos básicos de IA generativa simplifican los flujos de trabajo, mejoran la eficiencia y reducen los costes sanitarios, impulsado además la medicina personalizada, al permitir predecir el riesgo de enfermedades y sus resultados clínicos con mayor precisión. Así mismo, estos modelos de IA han mejorado la alfabetización en salud pública, empoderando a los pacientes al proporcionarles información sanitaria comprensible que les permite una mejor autogestión de sus enfermedades [3].

En los últimos años, el desarrollo de los grandes modelos de lenguaje (LLM, por sus siglas en inglés), como los transformadores pre-entrenados (GPT), BERT y el modelo de lenguaje Pathways (PaLM) ha tenido un gran impacto en diversos campos, entre los que se encuentran la generación de textos, la traducción automática y el diseño creativo de contenidos. Los LLM han revolucionado la interacción médico-paciente al simular las conversaciones humanas, lo que ha modificado los patrones de comunicación tradicionales en el campo de la salud [4]. Aunque los *chatbots* de IA pueden mejorar la alfabetización en salud, responder con rapidez a preguntas frecuentes sobre pruebas analíticas y ayudar a interpretar los resultados de las mismas, sus aplicaciones siguen encontrándose en fase inicial y los riesgos y dificultades asociados a los mismos precisan ser abordados [5], 6]. Cuando a los pacientes no se les proporciona con prontitud una explicación de los resultados de las pruebas analíticas, estos suelen recurrir a internet para buscar información, lo que puede derivar en desinformación y riesgos para la salud [7], [8], [9]. Además, el rendimiento de los diferentes modelos de *chatbot *varía significativamente, influenciado por factores como las configuraciones del modelo, la diversidad de las indicaciones y las metodologías de evaluación [10], [11], [12]. Los *chatbots* de IA tienen la capacidad de generar información aparentemente creíble pero imprecisa en realidad, por lo que es necesario que tanto los médicos como el personal del laboratorio sean conscientes de las ventajas y limitaciones de estas herramientas [13], 14].

El propósito de la presente revisión es realizar una síntesis de las ventajas y limitaciones de emplear *chatbots* en el laboratorio clínico, así como analizar las dificultades que esta práctica plantea y dar orientación para futuras investigaciones en el contexto del laboratorio clínico. Así mismo, proporcionamos algunas referencias de gran utilidad sobre el empleo de *chatbots*, dirigidas al personal de laboratorio, favoreciendo de esta forma la integración de la IA en la medicina.

## Necesidades y estado actual de la inteligencia artificial

Los informes de medicina de laboratorio son determinantes en la toma de decisiones clínicas, ya que influyen de manera sustancial en el diagnóstico y elección de tratamientos, jugando un papel crucial en el manejo de los pacientes. Sin embargo, la complejidad de dichos informes suele hacer que estos resulten confusos para los legos en la materia, llevando a no pocos pacientes a buscar información en los *chatbots* cuando presentan problemas de salud. Algunos estudios señalan que el 78 % de los usuarios de ChatGPT tienden a emplearlo para realizar un autodiagnóstico, lo que subraya la significativa demanda existente de fuentes fiables de información sanitaria [15]. Según el estudio realizado por Giardina et al., el 46 % de los pacientes hacen uso de las búsquedas en internet para interpretar los resultados de sus pruebas analíticas, lo cual también refleja la dificultad que estos encuentran a la hora de evaluar la gravedad de los resultados [5]. La ausencia de información clara en un plazo razonable de tiempo puede agravar la preocupación y dificultad de los pacientes para interpretar los resultados. Así mismo, el estudio realizado por Kopanitsa revela una mayor probabilidad de que los pacientes que reciben explicaciones generadas automáticamente presenten una mejor adherencia al seguimiento (71 %), en comparación con aquellos a los que únicamente se les proporcionan los resultados de las pruebas (49 %). Los pacientes suelen apreciar la prontitud de dichas aclaraciones, lo que evidencia el enorme potencial de los nuevos métodos de comunicación para mejorar notablemente la experiencia del paciente, así como los resultados clínicos [16].

Se han observado características jerárquicas significativas en la percepción y aceptación de la IA por parte del personal clínico [17], [18], [19]. La mayoría de los encuestados abogan por el uso de la IA como una herramienta complementaria, valorando especialmente su utilidad para el análisis de datos. En las autoevaluaciones, los médicos más jóvenes (<45 años) evidencian un menor conocimiento de la IA en comparación con sus colegas, si bien manifiestan una mayor disposición hacia el aprendizaje continuo. Por otro lado, el nivel de formación es determinante en los niveles de ansiedad, ya que los pacientes con estudios superiores manifiestan una menor preocupación por los errores de la IA, debido a su mayor capacidad para comprender aspectos técnicos.

Cabe señalar que el personal de laboratorio muestra una profunda preocupación por la pérdida de empleos (32 %), frente aquellos que ocupan puestos de dirección que presentan una menor inquietud al respecto (25 %). Los modelos multimodales de IA pueden mitigar las limitaciones de alfabetización en IA del personal de laboratorio integrando diversos tipos de datos (p.ej. imágenes morfológicas, biomarcadores celulares, y parámetros bioquímicos) en un marco analítico unificado [20]. Los sistemas multimodales son capaces de combinar redes neuronales convolucionales (CNN, por sus siglas en inglés) para extraer las características espaciales de los frotis de sangre, con modelos basados en grafos para analizar las redes de interacción celular y con algoritmos de regresión para interpretar las tendencias en los parámetros bioquímicos [21], 22]. Esta integración evitaría tener que sintetizar manualmente los datos, automatizando tareas de elevada complejidad, como la identificación de células displásicas en la leucemia, y determinando la correlación entre las anomalías morfológicas y las alteraciones en los parámetros bioquímicos (p.ej. niveles elevados de lactato deshidrogenasa). Para mejorar la accesibilidad, se pueden integrar estos modelos en plataformas sencillas con interfaces intuitivas que incluyan funciones como cargar imágenes arrastrándolas y soltándolas y la generación automática de informes obviando complejidades técnicas. Así mismo, la incorporación de componentes de IA (XAI, por sus siglas en inglés) como los mapas de calor que resaltan características celulares esenciales o los resúmenes en lenguaje natural de correlaciones bioquímicas, permite al personal de laboratorio validar los resultados sin que ello requiera un elevado nivel de conocimientos técnicos. Al agilizar los flujos de trabajo y proporcionar información contextualizada, los modelos multimodales salvan la brecha entre las capacidades de la IA y las tareas prácticas de laboratorio, reforzando la confianza y la adopción de esta tecnología incluso entre aquellos con una formación limitada en IA.

Actualmente, las plantillas de los laboratorios cuentan con una formación en IA alarmantemente insuficiente, habiendo demostrado unas competencias sólidas en IA solo el 10,8 % de estos profesionales, siendo la proporción aún menor entre los informáticos de los hospitales terciarios, en comparación con los centros sanitarios privados. Estas diferencias notables quedan evidenciadas por el hecho de que el 89,7 % de los encuestados haya declarado necesitar urgentemente formación en IA. Sin embargo, el 47,2 % de los laboratorios encuentran dificultades significativas a la hora de implementar con eficacia las tecnologías de IA, principalmente debido a la falta de equipos de asistencia técnica adecuados [23].

Existen discrepancias significativas entre los agentes implicados, relativas a la asignación de responsabilidades en los errores médicos relacionados con el uso de IA [24], [25], [26]. Las encuestas indican que el 66,7 % de los encuestados abogan por una responsabilidad compartida entre los usuarios y los fabricantes, frente a algunos que consideran que toda la responsabilidad por los productos con fallos de diseño debería recaer exclusivamente sobre el fabricante. Los médicos se inclinan por asignar una responsabilidad compartida entre los fabricantes y las instituciones sanitarias, afirmando que los fabricantes deberían ser los que asuman la principal responsabilidad si las herramientas de IA han sido sometidas a un proceso riguroso de validación y cumplen con los requisitos de calidad aplicables [26], 27].

## Integración de las interfaces HL7-FHIR/LIS y aspectos relacionados con la privacidad y la seguridad

Para conseguir una implementación práctica de los *chatbots* de IA en el laboratorio, es necesario integrarlos de forma sólida en la infraestructura existente, especialmente a través de interfaces estandarizadas como *Health Level 7 Fast Healthcare Interoperability Resources* (HL7-FHIR) y *Laboratory Information Systems* (LIS) [28], 29]. HL7-FHIR permite el intercambio interoperable de datos entre el modelo de IA y la base de datos del laboratorio, garantizando el acceso en tiempo real a los resultados estructurados de las pruebas, el historial de los pacientes, y los criterios diagnósticos [28], [29], [30], [31]. La interpretación de perfiles bioquímicos mediante la aplicación de la IA se podría mejorar a través de la comunicación bidireccional con los LIS, lo que permitiría generar alertas sobre valores críticos o realizar un análisis contextual de datos longitudinales [31]. Sin embargo, dicha integración requiere de la aplicación de robustos protocolos de ciberseguridad destinados a salvaguardar datos clínicos sensibles. Entre los requisitos básicos de confidencialidad y seguridad se encuentran el cifrado de extremo a extremo de las transmisiones de datos, controles de acceso por funciones que cumplan la legislación vigente, como el Reglamento General de Protección de Datos (RGPD) y la Ley de Portabilidad y Responsabilidad del Seguro Médico (HIPAA), así como la realización de auditorías periódicas destinadas a detectar posibles vulnerabilidades.

## Aplicación de *chatbots* a la inteligencia artificial en la medicina de laboratorio

Actualmente, el empleo de la IA se ha extendido a disciplinas como la radiología, para el reconocimiento de imágenes, si bien su utilización en la medicina de laboratorio se encuentra en fase inicial. Esto se debe principalmente al hecho de que la interpretación de los informes de laboratorio implica un enorme número de variables cuantitativas y cualitativas, tales como los síntomas, el historial médico, y los resultados analíticos [32], lo cual exige una aún mayor complejidad y precisión de los modelos de IA. Desde el lanzamiento de ChatGPT en 2022, este ha atraído el interés de la comunidad médica, habiéndose realizado importantes estudios sobre su rendimiento en los exámenes de habilitación médica, su viabilidad a la hora de responder a las preguntas de los pacientes, y su capacidad para ayudar a los médicos en la resolución de problemas clínicos, evidenciado su potencial aplicación en la medicina de laboratorio [33], [34], [35].

### Capacidades de ChatGPT en la medicina de laboratorio

Si bien no ha sido entrenado específicamente para procesar datos clínicos, se ha demostrado la viabilidad de emplear ChatGPT en la medicina. Munoz-Zuluaga et al. llevaron a cabo un estudio en el que le realizaron 65 preguntas a ChatGPT relativas a múltiples temas para evaluar sus capacidades y veracidad a la hora de responder a preguntas relacionadas con la medicina de laboratorio [36]. Según los resultados obtenidos, ChatGPT contestó correctamente al 50,7 % de las preguntas, dio una respuesta incompleta o parcialmente correcta al 23,1 % de las preguntas, ofreció información incorrecta o engañosa en el 16,9 % de las preguntas, y proporcionó una respuesta irrelevante al 9,3 % de las cuestiones. Cabe mencionar que las respuestas correctas solían referirse a preguntas relacionadas con conocimiento médicos o técnicos básicos (59,1 %), mientras que los errores se solían producir en las preguntas relacionadas con procedimientos o reglamentos sobre procedimientos de laboratorio (31 %). Aunque GPT-4 presenta mejoras significativas en relación a su veracidad, aún siguen siendo evidentes sus limitaciones en algunos campos. Girton et al. evaluaron la capacidad de ChatGPT para responder a 49 consultas reales realizadas por pacientes en plataformas de redes sociales como Reddit y Quora relativas a sus pruebas analíticas, y comparó las respuestas con aquellas proporcionadas por estudiantes de medicina [37]. Los revisores tendían a preferir las respuestas de ChatGPT por encima de las aportadas por los profesionales médicos. Este hallazgo subraya la evolución de ChatGPT a la hora de manejar cuestiones clínicas complejas, revelando así mismo carencias en la formación de los profesionales médicos en este campo. Aproximadamente la mitad de las respuestas de los estudiantes de medicina fueron calificadas como “inadecuadas”, frente a menos del 10 % de las respuestas de ChatGPT [37]. La escasa atención prestada a la medicina de laboratorio durante la formación médica podría contribuir a que incluso a los médicos más experimentados les resulte difícil proporcionar respuestas de calidad a preguntas reales.

Otro estudio comparó las respuestas de tres *chatbots* (ChatGPT, Gemini y Le Chat) con las proporcionadas por médicos colegiados en consultas *online* [38]. En términos generales, los *chatbots* fueron superiores a los médicos en las consultas *online* a la hora de interpretar los resultados del laboratorio. Aunque los médicos *online* quedaron en primer lugar en el 60 % de los casos, Gemini recibió una puntuación inferior en solo el 39 % de los casos. ChatGPT presentó una calidad y veracidad similares a los de los médicos humanos. Sin embargo, los *chatbots* mostraron una mayor tendencia a sobreestimar la gravedad clínica (incidencia del 22–33 %) frente a una tasa de sobreestimación de 1,0 % entre los médicos. Esto evidencia la dificultad de los *chatbots* a la hora de procesar información contextual compleja e interpretar datos analíticos, pudiendo la ausencia de patrones de referencia unificados dar lugar a interpretaciones incongruentes de los datos analíticos de los pacientes [38].

### Implementación de los *chatbots* para el diagnóstico en el laboratorio clínico

Los recientes avances en el campo de la inteligencia artificial han animado a la comunidad científica a analizar su capacidad para la interpretación de datos bioquímicos. Kaftan AN et al. demostraron la heterogeneidad en los niveles de veracidad de los modelos de IA (Copilot, Gemini) en el análisis de datos bioquímicos [39]. Copilot mostró un rendimiento superior en todas las métricas de evaluación, logrando ventajas estadísticamente significativas en la interpretación de parámetros bioquímicos, frente a otros modelos. Esto indica que su capacidad superior de procesamiento de datos radica en su sofisticada arquitectura algorítmica. Aunque el rendimiento de los otros modelos fue inferior al de Copilot, sus capacidades en campos especializados siguen haciendo de ellas herramientas útiles para su aplicación en tareas clínicas específicas [39].

La precisión diagnóstica y el rápido procesamiento de datos que la microbiología exige confieren a esta disciplina un valor estratégico especial a la hora de integrar la inteligencia artificial (IA) en la práctica clínica. La IA ha demostrado poseer una gran capacidad para mejorar la eficiencia en la toma de decisiones clínicas y optimizar el manejo de enfermedades infecciosas, redundando en mejores resultados clínicos. Sin embargo, es necesario seguir mejorando los sistemas de IA en el manejo de escenarios clínicos complejos y la generación de respuestas contextualmente apropiadas, con el fin de garantizar su relevancia clínica [40]. Cabe señalar que ChatGPT-4.0 presenta unas limitaciones notables a la hora de responder a consultas relacionadas con las pruebas de susceptibilidad a los antimicrobianos [41], 42]. De manera errónea, el modelo recomendó el uso de la clindamicina para las infecciones por enterococo y no incluyó las metodologías estándar para la evaluación de la susceptibilidad a la polimixina. Una información errónea podría inducir a errores en la práctica clínica, dando lugar a regímenes terapéuticos ineficaces y a un posible perjuicio para los pacientes.

Li Y et al. realizaron una evaluación sistemática del rendimiento de dos LLM, ChatGPT y Google Gemini, a la hora de abordar consultas clínicas relacionadas con el VHB [43]. ChatGPT-4.0 tuvo un rendimiento general superior, habiendo logrado una veracidad del 80,8 % en las preguntas basadas en la evidencia, frente a Google Gemini (73,1 %). El análisis por especialidad reveló una mayor utilidad diagnóstica de ChatGPT-4.0 en la interpretación serológica del VHB, si bien Google Gemimi proporcionó descripciones más completas de sus manifestaciones clínicas. Todos los modelos presentaron importantes limitaciones a la hora de ofrecer estrategias para la prevención de la infección por VHB, especialmente en lo relativo a la vigencia de la información sobre vacunas. Así, únicamente Google Gemini hizo referencia al consenso actualmente existente, por el cual se recomienda la vacunación neonatal contra la hepatitis B en las 12 horas posteriores al nacimiento.

Además, a pesar de las variaciones en la veracidad, tanto ChatGPT como Gemini incumplieron la instrucción de que el nivel de legibilidad de la respuesta estuviera adaptado un nivel de 8° grado (13/14 años) (Flesch-Kincaid 10,2–11,4 grado), lo que dificulta su comprensión por parte de los pacientes. Estos hallazgos subrayan la necesidad de que los resultados ofrecidos por los LLM sean verificados por un médico, con el objeto de garantizar tanto su interpretabilidad como la validez clínica. El presente estudio comparativo aporta información crucial sobre las ventajas e inconvenientes de los modelos en relación a su implementación en la clínica.

## Inconvenientes multidimensionales de los *chatbots* de salud en la práctica clínica

Aunque los *chatbots* fueron diseñados y presentados como herramientas conversacionales más que como herramientas para consultas sobre salud o de apoyo a la toma de decisiones clínicas, han demostrado poseer una capacidad asombrosa para detectar en interpretar alteraciones analíticas. Esta capacidad se evidencia en su eficacia para el procesamiento y análisis de grandes volúmenes de datos, una capacidad especialmente notable de los grandes modelos de lenguaje como ChatGPT. Sin embargo, dicho potencial se ve limitado por algunas deficiencias, especialmente porque estos modelos no han sido específicamente entrenados ni optimizados para su uso en la generación de informes de medicina de laboratorio.

### Dificultades en la aplicación de ChatGPT en la interpretación de datos analíticos

La evaluación del grupo de trabajo de la Federación Europea de Química Clínica y Medicina de Laboratorio (EFLM) revela importantes limitaciones en el uso clínico de ChatGPT a pesar de su capacidad para generar *“análisis de datos analíticos correctos en términos generales y relevantes para la seguridad”* [44]. Por otro lado, ChatGPT no identificó marcadores de enfermedad subclínica con valores en los límites de normalidad. Por ejemplo, no tuvo en cuenta que los niveles elevados de GGT no tienen por qué ser necesariamente indicativos de daño hepático, ni que una distribución normal de la subpoblación de leucocitos no garantiza la integridad del sistema inmunológico. Dicho análisis por parámetros corre el riesgo de pasar por alto indicadores de patología incipiente. Así mismo, en el reconocimiento de factores preanalíticos surgen deficiencias importantes. Aunque ChatGPT advierte correctamente del riesgo de diabetes ante la presencia de niveles elevados de glucosa y HbA1c, omite variables esenciales previas a la toma de muestras (p. ej. obtención en ayunas) a la hora de interpretar resultados discordantes (niveles normales de glucosa concurrentes a niveles normales de HbA1c).

Además, ChatGPT realiza una síntesis incorrecta de los perfiles multianalitos, y la interpretación de los marcadores de función hepática (ALT, AST, bilirrubina, GGT) no integra el contexto clínico, que resulta esencial a la hora de obtener un razonamiento diagnóstico completo [44]. Por otro lado, ChatGPT no analiza en profundidad los riesgos asociados a los resultados analíticos. Por ejemplo, en los casos de anemia severa o de alteraciones en el perfil lipídico, se limita a aconsejar a los pacientes que consulten a un médico, pero no les informa de la posible gravedad clínica de estas patologías, lo que podría poner en riesgo la salud de los pacientes. Por último, ChatGPT no es capaz de distinguir eficazmente entre los valores atípicos y los valores umbral críticos. Esta limitación podría derivar en errores médicos en escenarios clínicos críticos. La incapacidad para diferenciar entre valores anormales y valores clínicos que requieren atención médica urgente resulta especialmente preocupante, ya que dicha distinción resulta crucial para garantizar una intervención médica adecuada.

### Diversidad y variabilidad entre *chatbots*

En el ecosistema actual de *chatbots*, la significativa variabilidad entre los diferentes modelos dificulta considerablemente la realización de estudios comparativos. Los modelos de IA generativa están diseñados con diferentes arquitecturas, entornos y objetivos, por lo que su rendimiento y calidad a la hora de generar contenido difieren sustancialmente. Así, su arquitectura y capacidades influyen directamente en la veracidad y efectividad de sus resultados. En algunas tareas concretas, existen diferencias significativas en el rendimiento de los distintos modelos de IA generativa. Por ejemplo, se ha demostrado que Bing es el que presenta mayor veracidad y especificidad en la predicción de interacciones entre fármacos, superando significativamente a Bard y ChatGPT-4, lo que evidencia que algunos modelos podrían presentar algunas ventajas para aplicaciones específicas [45].

Así mismo, no debemos obviar la variabilidad del propio modelo, ya que el mismo modelo puede generar resultados incongruentes cuando se emplean diferentes configuraciones, datos de entrada o estrategias de generación, lo cual es especialmente relevante en el campo de la salud pública, donde dicha inconsistencia puede tener importantes implicaciones en la toma de decisiones [45], 46]. La heterogeneidad en el rendimiento de un modelo no afecta únicamente a la efectividad de la divulgación de la información, sino que también influye directamente en la experiencia y satisfacción del usuario. Por ejemplo, se ha demostrado que Bard proporciona información más comprensible sobre la rinoplastia, seguido de ChatGPT y Bing [47]. De este modo, tanto los investigadores como los usuarios deben ser cautelosos y seleccionar el modelo de IA generativa con mejor rendimiento en tareas concretas y que satisfaga las necesidades del propio usuario.

### Riesgos asociados a la actualización de las versiones de los *chatbots*

Aunque el fin último de las actualizaciones de los *chatbots* es mejorar sus capacidades y rendimiento, estas también plantean algunas dificultades relativas a la coherencia, calidad y fiabilidad de los datos. Las actualizaciones de los modelos pueden introducir nuevos conocimientos, con el riesgo de hacer referencia a datos obsoletos, especialmente en campos que evolucionan rápidamente, como es el caso de la medicina. Por ejemplo, los datos empleados para entrenar ChatGPT no incluían los últimos valores de referencia para el plomo en sangre de 3,5 μg/dL en los hemogramas de pacientes pediátricos. Por el contrario, CopyAI proporcionó valores de referencia exactos para el plomo en sangre, lo que indica que las diferencias en la actualización de datos de entrenamiento entre los distintos *chatbots* pueden derivar en resultados de impresión clínica diferentes [48]. Además, el rendimiento no siempre mejora con las actualizaciones, pudiendo disminuir su veracidad en algunas tareas. Un estudio realizado por las universidades de Stanford y California, Berkeley, reveló que si bien GPT-4.0 proporciona información más completa en algunas áreas, su veracidad en tareas como las matemáticas, la codificación y el razonamiento visual disminuyó [49]. Este fenómeno evidencia la importancia de evaluar continuamente el rendimiento de los LLM, especialmente en campos como la medicina de laboratorio, donde la precisión resulta crucial.

### Problemas de coherencia en la generación de contenidos aplicando modelos de IA generativa

La consistencia de los resultados de los *chatbots* en la generación de contenidos merece especial atención, especialmente en campos especializados como la medicina. Los estudios indican que pequeñas diferencias en la redacción de los *prompts* o en la información contextual pueden provocar variaciones significativas en el contenido generado, pudiendo afectar a su fiabilidad a la hora de darle una aplicación práctica a dicha información [10], 50], 51]. Un estudio llevado a cabo por Kochanek y col. reveló una consistencia del 85–88 % entre las respuestas de GPT-4.0 a la misma pregunta en el transcurso de cuatro días, lo que demuestra la influencia de la incertidumbre en la generación de contenidos [52]. En el campo de la medicina, donde es esencial que los resultados sean coherentes, la heterogeneidad en las respuestas podría plantear riesgos considerables. Es necesario continuar evaluando la reproducibilidad y precisión de ChatGPT en el campo de la medicina de laboratorio, con el fin de poder garantizar su fiabilidad como fuente de referencia en aplicaciones clínicas [37].

Además, los modelos de IA generativa se pueden ver influidos por sesgos culturales y socioculturales inherentes a los datos de entrenamiento empleados, lo que puede provocar incoherencias en el contenido generado, dependiendo del contexto cultural o lingüístico. Por ejemplo, Want y col. observaron que ChatGPT mostró un rendimiento significativamente superior en inglés que en chino durante el examen de habilitación en farmacia de Taiwán [53]. Por otro lado, Alfertshofer y col. determinaron que el rendimiento de ChatGPT varió sustancialmente en los exámenes de habilitación en medicina de seis países, lo que evidencia la influencia de los factores nacionales y lingüísticos [54]. Estos hallazgos indican la necesidad de realizar estudios en profundidad, así como de optimizar los modelos de IA generativa en entornos multilingües y multiculturales, con el fin de mejorar su adaptabilidad y precisión en distintos escenarios.

### Calidad limitada de la información sanitaria

Los *chatbots* están adquiriendo un papel cada vez más importante en la divulgación de información sanitaria, aunque la claridad, concisión y relevancia de sus respuestas deben ser contempladas con cautela [37]. En aras de la claridad, es preciso que el contenido generado sea fácil de entender, por lo que debe evitarse el empleo de jerga médica complicada para garantizar que la población general pueda entender fácilmente los puntos clave. A su vez, para garantizar la concisión de la información es necesaria una comunicación directa que no se extienda innecesariamente, lo que mejora la pertinencia de la información sanitaria y ayuda al público a mejorar su alfabetización en salud, permitiéndole así adoptar decisiones informadas sobre su salud. Por otro lado, resulta esencial que el contenido generado con IA sea relevante, ya que el acceso a información precisa y pertinente evita malentendidos, especialmente en escenarios en los que el paciente no puede preguntar a su médico en una consulta presencial, lo que evita situaciones de preocupación innecesaria o riesgos para la salud causados por la desinformación [55]. Dar prioridad a la relevancia de la información evita la sobrecarga de información, garantizando la emisión clara de mensajes importantes sobre salud. La información no relacionada con las consultas sobre salud puede dificultar la comprensión de las patologías por parte de la población, lo que agrava su confusión a la hora de manejar sus problemas de salud.

Sin embargo, la divulgación de información sanitaria a través de la IA debe establecer un equilibrio entre las diversas necesidades de los pacientes y los facultativos. Los pacientes suelen preferir respuestas breves y directas para entender información clave, lo que contrasta con la tendencia por parte de los médicos a ofrecer respuestas detalladas y exhaustivas. Esta divergencia de necesidades es una espada de doble filo: si bien la abundancia de información ayuda a los profesionales a realizar análisis en profundidad, puede sobrepasar al usuario, provocando dificultades de comprensión y preocupación. Independientemente del método de presentación empleado, es crucial que se proporcione información completa, ya que la ausencia de detalles necesarios puede llevar a un diagnóstico erróneo o a un autodiagnóstico inexacto, aumentando los riesgos para la salud.

A modo ilustrativo, los pacientes con una alfabetización en salud limitada pueden encontrar dificultades a la hora de interpretar los resultados analíticos con precisión, tales como identificar los niveles altos de colesterol en el perfil lipídico, lo que puede derivar en graves problemas de salud como un infarto de miocardio o un accidente cerebrovascular [44]. De este modo, mejorar la capacidad de los pacientes para entender los resultados de sus pruebas analíticas resulta de gran importancia.

### Riesgos derivados de las “alucinaciones” de los *chatbots*

El uso de *chatbots* para la divulgación de información sanitaria se está extendiendo, suscitando inquietud por el riesgo que puede conllevar la desinformación. Aunque las herramientas de IA como GPT presentan algunas ventajas a la hora de acceder a la información, su incapacidad para explicar sus procesos de toma de decisiones impide que se puedan identificar y corregir los sesgos o errores del propio modelo. En el campo de la medicina, la desinformación generada por la IA puede tener graves consecuencias, como un autodiagnóstico erróneo, atención médica postergada, la expansión de enfermedades potencialmente peligrosas, y la pérdida de confianza por parte de la población en los profesionales e instituciones sanitarias. Por ejemplo, GPT ha proporcionado descripciones inexactas de los dispositivos analíticos aprobados por la FDA para la determinación de la troponina de alta sensibilidad en el lugar de atención. Dichas “alucinaciones” no son incidentes aislados, lo que subraya las limitaciones de los modelos de IA generativa a la hora de garantizar la precisión de la información [56], 57]. De este modo, resulta vital poder asegurar la precisión, fiabilidad y veracidad de la información médica generada por la IA. A la hora de diseñar y entrenar los modelos de IA, los desarrolladores deben dar prioridad a este objetivo, dado que algunos estudios revelan que la generación de información imprecisa es un fenómeno muy extendido entre dichas herramientas.

### Implicaciones jurídicas y éticas del empleo de datos para el entrenamiento de *chatbots*

Las implicaciones jurídicas y éticas que conlleva la utilización de datos para el entrenamiento de *chatbots* durante el desarrollo de modelos de inteligencia artificial generativa son motivo de preocupación [58], [59], [60], [61]. Estos aspectos no están relacionados únicamente con los datos, sino también con su uso y aceptabilidad, lo que afecta directamente a la transparencia y credibilidad del modelo. La transparencia de los datos resulta de especial relevancia, ya que garantiza que tanto usuarios como investigadores conozcan los métodos empleados para la obtención y la utilización de datos, pudiendo así validar la fiabilidad e integridad científica del modelo. Así mismos, no debemos obviar los aspectos éticos, especialmente cuando se utilizan materiales y datos clínicos sujetos a derechos, dado que es imperativo garantizar la legalidad del proceso de obtención y utilización de datos, a la vez que se salvaguardan los derechos de privacidad de los proveedores de datos.

Los modelos de IA generativa como ChatGPT están entrenados con datos extraídos de internet, heredando inevitablemente los sesgos que dicha información contiene, lo que se refleja en las respuestas de estos modelos [62]. En diversos estudios, se han identificado sesgos en los LLM en relación al género, la raza y otros factores sociales, lo cual no solo puede derivar en respuestas poco justas, sino que también puede exacerbar las desigualdades sociales. Dichos sesgos están íntimamente relacionados con aspectos como las lagunas de datos, tamaños muestrales insuficientes, y los sesgos inherentes a los datos básicos [63]. Si los datos de entrenamiento son más antiguos, se pueden ver amplificados los posibles sesgos y errores en las respuestas de los modelos [64]. Este fenómeno se torna especialmente evidente en los artículos existentes, especialmente en áreas clave como el diseño aleatorizado, lo que evidencia los posibles sesgos en los procesos de selección de las consultas.

## La Ley de IA de la UE y la *Guía de apoyo a la toma de decisiones clínicas* de la FDA

La Ley de Inteligencia Artificial de la Unión Europea (Ley IA), vigente desde el 12 de julio de 2024, constituye el primer marco jurídico regulatorio integral de la IA, y prioriza el desarrollo ético, la protección de los derechos fundamentales, y la innovación centrada en las personas [65]. Adoptando un enfoque basado en los riesgos, la Ley clasifica los sistemas de IA en cuatro niveles: riesgo inaceptable (prohibido); riesgo elevado (p.ej. diagnóstico médico, sujeto a estrictos criterios de seguridad, transparencia y responsabilidad); IA general con riesgos sistémicos (obligación de transparencia); y riesgo bajo o sin riesgo (regulación mínima) [66]. En el ámbito de la salud, la estricta normativa sobre el uso de IA de riesgo elevado en contextos clínicos exige la realización de pruebas rigurosas, la mitigación de sesgos, y el seguimiento post-comercialización, aunque siguen persistiendo algunas lagunas en relación a los derechos de los pacientes y la responsabilidad sobre las aplicaciones de riesgo bajo. Entre las dificultades, se encuentran una implementación desigual entre los estados miembro, marcos de responsabilidad civil ambiguos, y limitaciones cambiantes a la hora de detectar contenido generado con IA [67]. La Ley también aborda aspectos relacionados con los derechos de protección de la IA, exigiendo transparencia sobre la utilización de datos de entrenamiento y salvaguardando la propiedad intelectual, aunque exceptúa la investigación anterior a la comercialización [66]. Se espera que esta Ley tenga un impacto en otros territorios similar al que tuvo la RGPD, remodelando las prácticas de IA a nivel mundial, y haciendo precisa una formación en ética de la IA. Con el fin de establecer un equilibrio entre la regulación y la innovación, la Ley de IA promueve el desarrollo de una IA fiable, pero exige unas guías más claras, la colaboración de todos los agentes implicados, y la implementación de normas sanitarias adaptadas, con el fin de garantizar el acceso a la IA en condiciones de igualdad y seguridad para los pacientes.

Hasta la fecha, la FDA no ha aprobado ningún modelo de LLM para la toma de decisiones clínicas. Se han realizado algunos estudios para evaluar si los LLM se pueden adherir a las regulaciones en el contexto de las urgencias médicas. Los resultados obtenidos revelan que, aunque las recomendaciones sobre prevención proporcionadas por los LLM son adecuadas, el 100 % de las respuestas de GPT-4 y el 52 % de las respuestas de Llama-3 no se ajustaban a un escenario de urgencias, ofreciendo un soporte comparable al de un dispositivo, al sugerir diagnósticos y tratamientos concretos [68]. Aunque los *prompts* estuvieran basados en la guía de la FDA, no se logró que se ajustara a ella de manera uniforme. Estos resultados evidencian la necesidad urgente de desarrollar nuevos marcos regulatorios que aborden la problemática que el empleo de IA generativa plantea en el ámbito de la salud, dado que las guías actuales resultan insuficientes a la hora de evitar que los LLM ofrezcan de manera no autorizada resultados que corresponderían a los de un dispositivo médico autorizado.

## Establecimiento de un marco integral de evaluación para abordar las limitaciones de los *chatbots* de IA en el campo de la salud

A la luz de los riesgos que plantea el uso de *chatbots* de IA en la medicina, es imperativo desarrollar un marco integral de evaluación. El primer paso para garantizar la calidad de los resultados generados por la IA consiste en establecer unos criterios estrictos de evaluación que aborden la precisión, fiabilidad y relevancia de la información. Además, la evaluación periódica del rendimiento de los modelos permite identificar fallas del sistema, garantizando que todos los modelos de IA sean coherentes con la información más reciente en el campo de la medicina. Los mecanismos de retroalimentación, que reúnen información proporcionada por usuarios y profesionales, aportan datos de gran utilidad para la mejora continua de los modelos. La inclusión de mecanismos de revisión profesionales resulta igualmente crucial. La participación de médicos expertos mejora aún más la fiabilidad e integridad científica de los sistemas de IA, lo que garantiza que la información generada se ajuste a los criterios de ética y práctica médica.

Para tal fin, se han desarrollado diversas herramientas y guías para facilitar la evaluación y mejora de la calidad de la información en salud [69], [70], [71]. Sin embargo, las herramientas actuales de evaluación de información sanitaria no han sido adaptadas para evaluar específicamente la calidad de la información en salud generada por los modelos de IA. Para poder garantizar la fiabilidad y precisión de la IA en el campo de la medicina de laboratorio, es preciso disponer de herramientas de evaluación estandarizadas. Actualmente, las herramientas “METRICS’ y “CLEAR’” cuentan con amplia aceptación como métodos eficaces para evaluar información en salud generada mediante IA [72], 73].

La lista de comprobación METRICS proporciona un marco para diseñar y comunicar estudios estandarizados sobre IA, abarcando nueve temas clave: Modelo, Evaluación, Temporalidad, Rango, Aleatorización, Recuento, y Especificidad de las indicaciones y el lenguaje. Estos temas están orientados a analizar de manera exhaustiva el diseño y estado operativo de los modelos de IA, garantizando el rigor científico y la solidez de sus resultados. Por otro lado, la herramienta CLEAR se centra en la evaluación sistemática de múltiples dimensiones de contenido generado con IA, entre los que se incluyen cinco criterios clave: Integridad del contenido, Ausencia de información falsa en el contenido, Evidencia que respalde el contenido; Pertinencia del contenido, y Relevancia. En la Tabla 1 se detalla el significado concreto de cada criterio, mientras que la Tabla 2 ofrece a los evaluadores una guía clara para garantizar que el contenido generado con IA cumple unos criterios de calidad predefinidos.

**Tabla 1:** Entradas en METRICS.

Tipo de herramienta	Contenido del ítem	Limitaciones	Clasificación en la escala
METRICS incluye nueve ítems:			
1) Modelo	1) Modelo: La herramienta de IA generativa empleada en el artículo incluido debe mencionar explícitamente los ajustes exactos de cada herramienta.	1) Alcance limitado de la literatura: La literatura se obtuvo empleando una sola palabra clave, lo que puede derivar en omisiones.	Los items de METRICS se puntuaron en una escala de Likert de 5 puntos: 5=excelente, 4=muy buena, 3=buena, 2=satisfactoria, y 1=subóptima.
2) Evaluación	2) Evaluación: La estrategia para evaluar la calidad del contenido equilibra la objetividad (hallazgos sin sesgos) y la subjetividad.	2) Sesgo de la base de datos y del lenguaje: La literatura en inglés de las bases de datos como posibles sesgos de selección. Scopus, PubMed y Google Scholar puede introducir	
3) Temporalidad	3) Temporalidad: El momento exacto y duración de las pruebas del modelo de IA.	3) Temas insuficientes: Debido a las limitaciones del equipo de autores (contexto de una sola disciplina), se pueden haber omitido temas interdisciplinares relevantes.	
4) Transparencia	4) Transparencia: Transparencia de las fuentes de datos (incluidas los permisos de los contenidos sujetos a derechos).	4) Riesgo de subjetividad: La selección de temas y evaluación de los autores	
5) Rango	5) Rango: El ámbito de los temas probado (uno/varios temas relacionados/temas no relacionados; amplitud de las consultas intratemáticas e intertemáticas).	5) Igual ponderación en la puntuación: Todos los METRICS adoptaron la misma ponderación, independientemente de las diferencias en importancia entre los distintos indicadores.	
6) Aleatorización:	6) Aleatorización: Grado de aleatorización en la selección de temas para mitigar sesgos.	Limitaciones de cobertura de los modelos: El estudio se centró en ChatGPT, Bing y Bard, sin tener en cuenta otros modelos generativos (como Claude).	
7) Factores particulares	7) Persona: Papel subjetivo en la evaluación de contenidos y fiabilidad interevaluador (concordancia/discordancia).		
8) Recuento	8) Recuento: Número de consultas ejecutadas por modelo (tamaño muestral).		
9) Especificidad de los *prompts* y del idioma	9) Especificidad: *Prompts* (instrucciones: Formulación exacta, mecanismos de retroalimentación y bucles de aprendizaje. Idioma: idioma(s) empleado en las pruebas, incluyendo aspectos culturales (ej. Adecuación cultural y lingüística).		

**Tabla 2:** Entradas en CLEAR.

Tipo de herramienta	Contenido del ítem	Limitaciones	Clasificación en la escala
CLEAR incluye cinco ítems:			
1) Integridad del contenido	1) ¿Es suficiente el contenido? Por integridad se entiende que la información se genera de manera optimizada, no siendo ni excesiva ni deficiente.	1) Limitaciones en el tamaño muestral: La evaluación con la herramienta CLEAR se basa exclusivamente en un pequeño grupo homogéneo de profesionales sanitarios, lo que provoca sesgos; los datos de prueba incluyen afirmaciones generadas artificialmente, y carecen de validación contextual.	Los ítems de CLEAR se puntuaron en una escala de Likert de 5 puntos: 5=excelente, 4=muy buena, 3=buena, 2=satisfactoria, y 1=subóptima.
2) Ausencia de información falsa en el contenido	2) ¿Es el contenido preciso? La generación de información en salud incorrecta con herramientas de IA puede tener consecuencias negativas.	2) Validación insuficiente de la herramienta: La herramienta CLEAR no se ha comparado con otras herramientas de evaluación de la información, como DISC, para identificar sus fortalezas y limitaciones; se debe seguir analizando la herramienta para confirmar su efectividad probándola en relación a temas de salud más amplios, especialmente aquellos que son más controvertidos.	
3) Evidencia que respalde el contenido	3) ¿Está el contenido basado en la evidencia? Esto significa que la información en salud proporcionada por el modelo de IA se debe basar en los últimos avances científicos y evitar sesgos, desinformación o información falsa.	3) Impacto de la dinámica del modelo de IA: los modelos de IA se actualizan e iteran continuamente (p.ej. los cambios de GPT-4 a GPT-5 pueden arrojar resultados diferentes con la misma consulta con el transcurso del tiempo); el rendimiento del modelo es sensible al diseño de los *prompts*, y las variaciones de los prompts pueden provocar discrepancias en los resultados.	
4) Pertinencia del contenido	4) ¿es el contenido claro, conciso y fácil de entender? El contenido también debe proporcionar una explicación clara y bien organizada en siguiendo una secuencia lógica para facilitar su comprensión.	4) Generalización limitada: Las conclusiones actuales se basan en evaluaciones realizadas en una especialidad concreta, por lo que es necesaria su validación por expertos de distintos ámbitos (como desarrolladores de IA y representantes de pacientes).	
5) Relevancia	5) ¿Omite el contenido información irrelevante? Por irrelevancia se entiende la necesidad de proporcionar información en salud precisa y pertinente.		

Malik Sallam et al. evaluaron tres modelos de IA generativa (ChatGPT, Microsoft Bing, y Google Bard) mediante el empleo de la herramienta METRICS [72]. La puntuación total media de los tres modelos según METRICS fue de 3,0 (SD 0,58). Al analizar los criterios por separado, el criterio “Modelo” fue el que recibió una puntuación media más alta, seguido de “Especificidad”. Por otro lado, los criterios que obtuvieron puntuaciones más bajas fueron “Aleatorización” (clasificada como subóptima) y “Factores individuales” (clasificado como satisfactorio). En otro estudio, los cinco modelos de IA se evaluaron por separado empleando la herramienta CLEAR en relación a cinco temas diferentes [73]. Microsoft Bing fue el que obtuvo una puntuación CLEAR más alta (media 24,4±0,42), seguido de ChatGPT-4 (media: 23,6±0,96), Google Bard (media: 21,2±1,79), y finalmente de ChatGPT-3.5 (media: 20,6±5,20).

**Figura 1:**
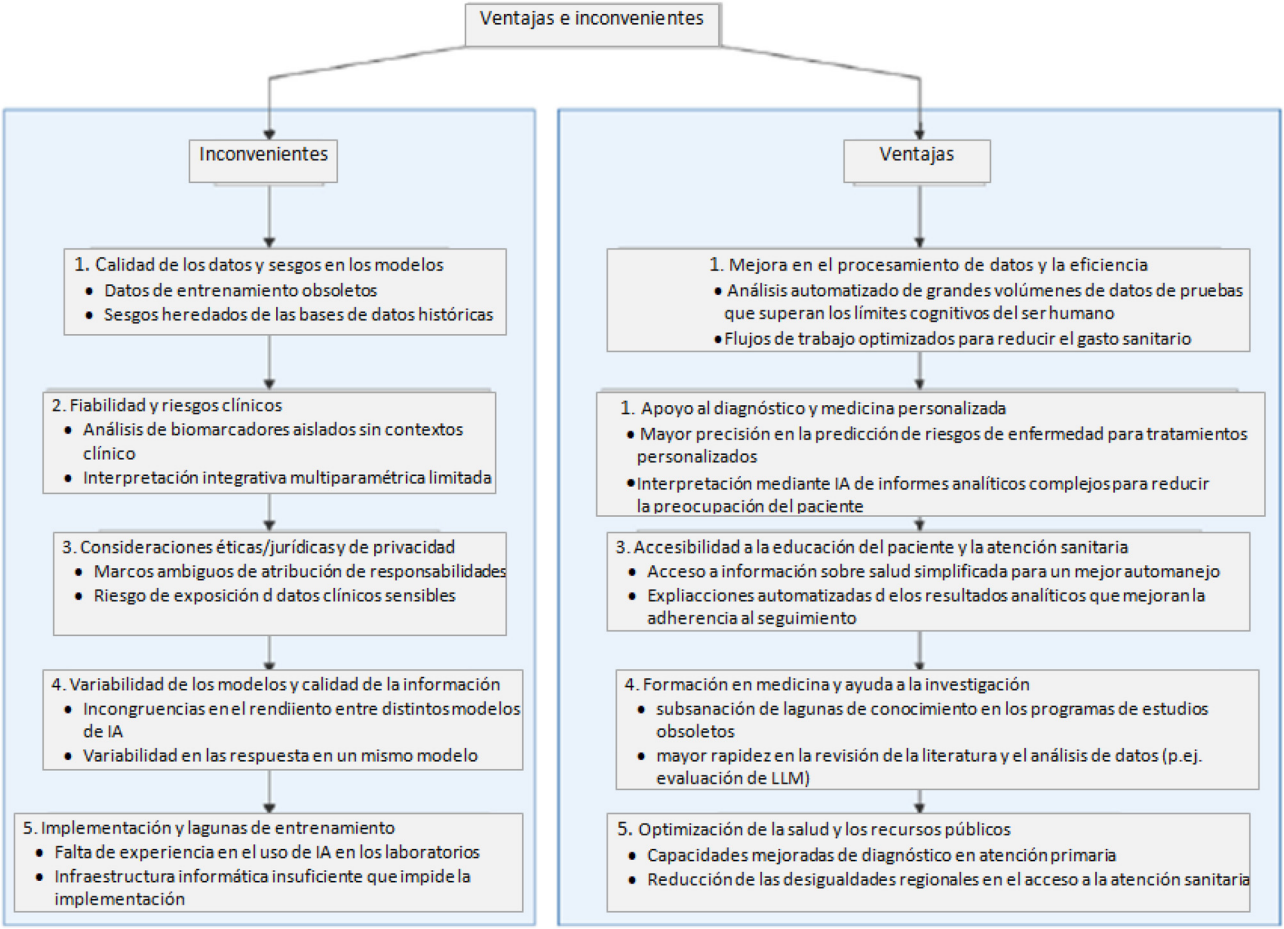
Diagrama de vantajes e inconvenientes.

Si bien METRICS y CLEAR han facilitado la evaluación de contenidos generados con IA, también presentan algunas limitaciones (detalladas en las tablas 1 y 2). Por ejemplo, es posible que estas herramientas no abarquen todas las variables en contextos concretos o tengan una aplicación limitada en algunos campos especializados. Estas limitaciones precisan ser validadas en estudios que confirmen su efectividad y adecuación para su aplicación en la práctica. La identificación y resolución de dichas limitaciones nos permitiría mejorar la credibilidad de la información en salud generada con IA, lo que contribuiría a establecer una base sólida para la toma de decisiones clínicas (Figura 1).

La formación en medicina es un proceso que requiere tiempo y se actualiza lentamente, lo que dificulta que los estudiantes asimilen todos los aspectos esenciales. De este modo, los *chatbots* de IA presentan gran potencial como importantes herramientas auxiliares en la formación médica, lo que contribuye a cubrir lagunas en la adquisición de conocimientos. Sin embargo, la resolución creativa de problemas sigue siendo una capacidad exclusiva del ser humano. Para aprovechar al máximo el potencial de la IA en la medicina, resulta esenciar contar con la colaboración de los desarrolladores de IA y los profesionales de la medicina.

La medicina de laboratorio es un campo en rápida evolución, ya que surgen continuamente nuevas tecnologías y métodos analíticos que se van integrando en la práctica clínica. Si resolviéramos eficazmente las limitaciones de los *chatbots* de IA, estos se convertirían en herramientas indispensables para los facultativos y los técnicos de laboratorio en la práctica rutinaria. Superar las dificultades existentes podría redundar en una mayor precisión diagnóstica, una mejor calidad en la atención a los pacientes, y en la promoción de la medicina personalizada. La colaboración entre la IA y los médicos expertos humanos sentará las bases para el futuro desarrollo de la medicina de laboratorio.
